# OR13-003 - TNFRSF11A molecular defects cause autoinflammatory disorders

**DOI:** 10.1186/1546-0096-11-S1-A265

**Published:** 2013-11-08

**Authors:** I Jéru, E Cochet, P Duquesnoy, V Hentgen, B Copin, M Mitjavila-Garcia, S Sheykholeslami, G Le Borgne, F Dastot, S Karabina, M Mahevas, S Chantot-Bastaraud, L Faivre, S Amselem

**Affiliations:** 1 UMR_S933, INSERM, France; 2 Université Pierre et Marie Curie-Paris, France; 3 Génétique et d’Embryologie Médicales, Hôpital Trousseau, Paris, France; 4 Centre de Référence des Maladies AutoInflammatoires (CeRéMAI), Centre Hospitalier de Versailles, Le Chesnay, France; 5 U.935, INSERM, Villejuif, France; 6 Médecine Interne, Hôpital Henri Mondor, Créteil, France; 7 Centre de Génétique et Centre de Référence Anomalies du Développement et Syndromes Malformatifs, Hôpital d’Enfants, Dijon, France

## Introduction

Hereditary recurrent fevers (HRF) are autoinflammatory disorders whose etiology remains unknown in many cases.

## Objectives

To identify a new HRF gene

## Methods

Comparative genomic hybridization (CGH, 385K array) was performed in the proband. *TNFRSF11A* was screened by Sanger sequencing in other patients. *TNFRS11A* expression was quantified by fluorescence-activated cell sorter analysis (FACS). NF-k B activation was assessed using a luciferase assay in HEK293 cells transfected with plasmids encoding wild-type and mutated TNFRSF11A.

## Results

Array-CGH analysis performed in a patient with multiple congenital anomalies and a recurrent fever syndrome revealed a de novo heterozygous chromosomal rearrangement encompassing a duplication of *TNFRSF11A*. This transmembrane receptor binds the TNFSF11 cytokine, activates NF-k B signaling, and regulates fever in rodents, consistent with a possible role in HRF. *TNFRSF11A* screening in other patients with genetically-unexplained HRF revealed a heterozygous frameshift mutation in a patient and her affected mother. The mutated protein is expressed at similar levels as the normal receptor on leukocytes. Most importantly, this mutation results in a gain of function on NF-k B signaling, since the mutated protein is more responsive to TNFSF11 stimulation than the wild-type receptor. Since *TNFRSF11A* (also known as *RANK*) was previously known for its key role in osteoclastogenesis, the medical history of our patients was reassessed and revealed minor symptoms also found in patients with *TNFRSF11A*-associated bone disorders.

## Conclusion

The implication of *TNFRSF11A* in HRF reveals a key role of this receptor in autoinflammation and opens up new fields of research at the crossroads between bone metabolism and innate immunity.

## Disclosure of interest

None declared

